# Supplementary material for: Ibero-American Consensus Review and Incorporation of New Biomarkers for Clinical Practice in Colorectal Cancer
Source: Cancers (Basel). 2023 Sep 1;15(17):4373. doi: 10.3390/cancers15174373 (PMC10487247; doi:10.3390/cancers15174373)
Supplement: Supplementary file 1 [file cancers-15-04373-s001.zip › cancers-2428137-supplementary.pdf]

Table S1. Prognostic and predictive value of each biomarker

Biomarker	Prognostic value	Predictive value	Determination recommendations
RAS	• Mutations in RASc confer adverse prognosis [13-15], especially p.G12C/p. G12S mutations [16].<br>• They are associated with unfavorable prognosis in patients with mCRC who undergo metastasectomy [17-19].	• The presence of RAS mutations is a strong predictor of non-response to anti-EGFR [1,20,21].<br>• In RAS WT population, and especially in left-sided tumors BRAF WT, the addition of anti-EGFR to chemotherapy vs. chemotherapy alone is associated with better OS, PFS, ORR. In mutated RAS patients, anti-EGFR is detrimental [22-30].<br>• Clinical trials evaluating chemotherapy + anti-EGFR vs. chemotherapy + bevacizumab have confirmed the negative impact of RAS mutations to anti-EGFR [2,31,32].	• RAS mutation testing is recommended in patients with mCRC at the time of diagnosis, which allows defining prognosis and treatment, especially the use of anti-EGFR (and in the future RAS targeted therapy) [1,20].<br>• In RAS WT disease that has received anti-EGFR antibodies and shows progression, and maintenance or rechallenge to anti-EGFR is considered, re-analysis of RAS mutations is recommended [33].<br>• Mutational analysis should include HRAS, KRAS and NRAS, exons 2 (codons 12 and 13), 3 (codons 59 and 61) and 4 (codons 117 and 146) in tissue or in liquid biopsy [1,34].<br>• It is not routinely recommended in non-metastatic patients [12].
BRAF	• BRAF V600E mutation is a potent adverse prognostic biomarker associated with short survival times [9,35,36].<br>• Other BRAF mutations (not V600E) are associated with MSI and better OS compared to BRAF V600E mutation [20].	• It is a predictor of non-response to anti-EGFR [20,21,34,36], except when used together with BRAF inhibitors, as the combination appears synergistic [37].	• BRAF mutation testing is recommended in patients with mCRC in tissue at diagnosis for prognostic stratification [34,36], and to define treatment [10,11,36].<br>• In tumors with dMMR, BRAF determination helps to evaluate the

			presence of Lynch syndrome. BRAF V600E mutation is consistent with sporadic cancer [34].
MSI	• In stages II–III of mCRC, MSI is related to a better prognosis, and specifically in stage II. The better prognosis occurs in the absence of benefits from adjuvant treatment with fluoropyrimidines [1].<br>• In stage IV, MSI is associated with an unfavorable prognosis, most likely due to its association with BRAF mutations [1].	• It is a strong predictor of good response to immune checkpoint inhibitors [38,39].	• MSI analysis at diagnosis is recommended for all patients with advanced CRC, as it has an important prognostic and predictive role. It allows identifying a group of patients who benefit from anti PD-1 immunotherapy [1].<br>• In patients with stage II CRC, MSI predicts low benefit of adjuvant fluoropyrimidine monotherapy. The ASCO guideline recommends that in the absence of risk factors, no adjuvant should be given to patients with MSI, and in those with risk factors, fluoropyrimidine + oxaliplatin [40].<br>• In patients with stage III CRC, its prognostic and predictive value is less clear [1]. In these cases its detection is limited to identifying Lynch syndrome [12].
PD-L1	• PD-L1 expression in tumor cells is an adverse prognostic factor associated with worse OS [20,41-43] and worse PFS [41].<br>• It has been reported that the adverse prognostic role of PD-L1 expression	• The predictive role of PD-L1 in mRCC is not yet clear.<br>• In the Checkmate-142 trial involving dMMR/MSI-H patients, PD-L1 expression had no correlation with	• There are no data to date to recommend the routine determination of PD-L1 in patients with mCRC. For now, its use should be limited to the research setting.

	appears to be limited to cases of high coexpression of PD-1 in TILs (only reported in patiernts with dMMR or MSI-H [44].	response rates and disease control with immunotherapy treatment [45].<br>• The MODUL study also found no correlation between PD-L1 expression and response to fluoropyrimidine + bevacizumab ± atezolizumab in BRAF WT mCRC [46].	
PI3K	• Secondary mutations in PI3KCA were associated with tumor resistance to treatment with EGFR+BRAF inhibitors for BRAF V600E mutant cancer [47].		• PIK3CA determination is only recommended for inclusion of patients in a clinical trial.<br>• It could play a potential role in chemoprevention.
FGFR	• FGFR2 overexpression in CRC is associated with tumor progression.<br>• High FGFR2 expression is associated with patient survival. FGFR2 has been associated with PDL-1 upregulation via the JAK/STAT3 pathway [48].		• There are insufficient data to recommend its determination in mCRC in practice, except in the context of clinical research.
HER2	• HER2 amplifications are typically associated with EGFR-targeted therapy resistance [49].<br>• As in other tumor models, HER2-L (HER2-low: IHC 1+/IHC 2+ FISH not amplified) expression is being investigated. Better prognosis and associations with RAS mutations have been described [50,51].		• Its determination is recommended in patients with mCRC as a predictive factor for anti EGFR therapy (fist-line), and in patients with left mCRC and RAS WT with progression to anti EGFR therapy, with anti-HER2 therapeutic criteria.

NTRK			• ESMO recommendations for using NGS recommend including NTRK testing [52].<br>• According to local Guidelines, regarding enriched population for determination, MSI-H, RAS WT with progression to standard therapy.
RET			• Study in patients with advanced CRC, MSI-H, RAS WT, with resistance to immunotherapy.
ALK	• ALK, ROS1, or NTRK rearranged tumors seems to have an independent poor prognosis [53].<br>• ALK, ROS, and NTRK rearranged tumors seem not to derive benefit from anti-EGFR monoclonal antibodies, thus confirming preclinical observations [54,55].		• For its study, genomic profiling and NGS are recommended. Another platform should be used depending on its availability and cost-effectiveness.
ROS1	• No clinical evidence of ROS1 inhibitors.	• No clinical evidence of ROS1 inhibitors.	• Study in patients with advanced CRC, MSI-H, RAS WT, with resistance to immunotherapy.<br>• For its study, genomic profiling and NGS are recommended. Another platform should be used depending on its availability and cost-effectiveness.
NRG1	• High NRG1 expression by IHC or FISH was associated with lower PFS [56].		• Approved for tumor-agnostic treatment (not in Latin America).<br>• Determination only under clinical trial.

MET	• MET amplification is recognized as a potential mechanism of acquired resistance for mCRC treated with anti-EGFR therapy [57].<br>• c-MET overexpression in CRC have showed a poor prognostic factor [58,59].		• Study of amplification or mutation in cases of disease resistant to EGFR inhibitors, prognostic role and/or eventual study for participation in a clinical trial.
WEE1	• Overexpression of WEE1 by RT-qPCR and IHC (nuclear 2+ or 3+) in metastatic CRC correlates with a worse prognosis [60].		• Recent evidence from a phase II study with adavosertib [61]. Requires validation for its determination as a predictive factor.<br>• Only in the context of clinical trials.
HRD	• In the TRIBE2 study, patients with MSS/pMMR tumors and HRD showed longer OS compared with MSS/dMMR tumors without HRD [62].		

ASCO: American Society of Clinical Oncology; dMMR: DNA mismatch repair-deficiency; DSB: DNA double-strand breaks; ESMO: European Society for Medical Oncology; FISH: fluorescence in situ hybridization; HRD: homologous recombination deficiency; IHC: immunohistochermistry; mCRC: metastatic colorectal cancer; MSI: microsatellite stability; MSS: microsatellite stable; MSI-H: microsatellite stability high; NGS: next-generation sequencing; OS: overall survival; ORR: objective response rate; PARP: poly-ADP ribose polymerase; PFS: progression-free survival; TIL: tumor-infiltrating lymphocyte; WT: wild type.
